# Feasibility and relevance of compound strain imaging in non-stenotic arteries: comparison between individuals with cardiovascular diseases and healthy controls

**DOI:** 10.1186/s12947-017-0104-9

**Published:** 2017-05-18

**Authors:** Martijn F.H. Maessen, Thijs M.H. Eijsvogels, Ayla Grotens, Maria T.E. Hopman, Dick H.J. Thijssen, Hendrik H.G. Hansen

**Affiliations:** 1 Department of Physiology, Radboud university medical center, Nijmegen, The Netherlands; 2 Department of Radiology and Nuclear Medicine, Radboud university medical center, Medical UltraSound Imaging Center (MUSIC), P.O. Box 9101 (766), 6500 HB Nijmegen, The Netherlands; 3 Research Institute for Sports and Exercise Sciences, Liverpool John Moores University, Liverpool, UK

**Keywords:** Strain imaging, Myocardial infarction, Ultrasound imaging, Cardiovascular assessment, Intima media thickness

## Abstract

**Background:**

Compound strain imaging is a novel method to noninvasively evaluate arterial wall deformation which has recently shown to enable differentiation between fibrous and (fibro-)atheromatous plaques in patients with severe stenosis. We tested the hypothesis that compound strain imaging is feasible in non-stenotic arteries and provides incremental discriminative power to traditional measures of vascular health (i.e.*,* distensibility coefficient (DC), central pulse wave velocity [cPWV], and intima-media thickness [IMT]) for differentiating between participants with and without a history of cardiovascular diseases (CVD).

**Methods:**

Seventy two participants (60 ± 7 years) with non-stenotic arteries (IMT < 1.1 mm) were categorized in healthy participants (CON, *n* = 36) and CVD patients (*n* = 36) based on CVD history. Participants underwent standardised ultrasound-based assessment (DC, cPWV, and IMT) and compound strain imaging (radial [RS] and circumferential [CS] strain) in left common carotid artery. Area under receiver operating characteristics (AROC)-curve was used to determine the discriminatory power between CVD and CON of the various measures.

**Results:**

CON had a significantly (*P* < 0.05) smaller carotid IMT (0.68 [0.58 to 0.76] mm) than CVD patients (0.76 [0.68 to 0.80] mm). DC, cPWV, RS, and CS did not significantly differ between groups (*P* > 0.05). A higher CS or RS was associated with a higher DC (CS: *r* = −0.32;*p* < 0.05 and RS: *r* = 0.24;*p* < 0.05) and lower cPWV (CS: *r* = 0.24;*p* < 0.05 and RS: *r* = −0.25;*p* < 0.05). IMT could identify CVD (AROC: 0.66, 95%-CI: 0.53 to 0.79), whilst the other measurements, alone or in combination, did not significantly increase the discriminatory power compared to IMT.

**Conclusions:**

In non-stenotic arteries, compound strain imaging is feasible, but does not seem to provide incremental discriminative power to traditional measures of vascular health for differentiation between individuals with and without a history of CVD.

## Background

Atherosclerosis is a multifactorial disease, which affects the vasculature and usually develops in humans over the course of years to decades [1]. Early detection of people at risk for CVD is of paramount importance to prevent life threatening events, such as stroke or myocardial infarction. Results from large cohort studies [2, 3] indicate that several vascular markers obtained via ultrasound techniques, such as the distensibility coefficient (DC), central pulse wave velocity (cPWV), or intima-media thickness (IMT), improve cardiovascular risk stratification. Recent studies also investigated the performance of carotid artery wall strain parameters by commercially available speckle tracking techniques for CVD risk stratification [4–6]. Park et al. observed a significant correlation between circumferential strain, the amount of cardiovascular risk factors and the Framingham Risk Score [4]. Catalano et al. found that peak systolic circumferential strain adjusted for pulse pressure could differentiate between three cardiovascular risk groups, whereas for instance IMT could not [6]. Although these results are very promising, these commercial techniques were initially developed for myocardial strain estimation. All commercially available packages for myocardial strain estimation use either B-mode data or the envelope signal of the raw radiofrequency data for tracking. [7] It is known that more accurate strain estimation can be performed when using the underlying raw ultrasound radiofrequency (RF) data, because it contains the phase information of the ultrasound signal [8, 9]. Recently, several RF-based strain techniques specifically designed to evaluate arterial wall strain have been developed [10–15]. Some of these techniques have already been validated against post-endarterectomy histology and/or magnetic resonance imaging derived plaque composition [16–19]. In all of these validation studies patients with severe amounts of stenosis (>50%) were included. Whether these RF-based techniques are also feasible in non-stenotic arteries and provide information that can discriminate between healthy, asymptomatic subjects and those with a history of cardiovascular disease, needs to be explored. Despite the promising findings for speckle-tracking-based strain analysis, it is also still unclear if the information provided by RF-based strain imaging has additional value for cardiovascular risk stratification over traditional measures of conduit arteries, such as cPWV or IMT.

In this study, we tested whether compound strain imaging, an in house built and validated RF-based strain imaging technique designed for transverse imaging planes, is feasible in non-stenotic arteries and provides information that has additional value compared to traditional measures of conduit arteries (i.e., cPWV, DC or IMT) for discrimination between participants with and without a history of CVD.

## Methods

### Participants

A total of 72 male participants aged 46 to 77 years with non-stenotic arteries were included. Based on CVD history the participants were categorized in healthy, asymptomatic controls (CON, *n* = 36) and CVD patients (CVD, *n* = 36). CVD participants suffered from a myocardial infarction in the past and used cardiovascular medication (anticoagulants, antihypertensive, or lipid lowering agents). Smokers and diabetics were excluded from the study. The Local Committee on Research Involving Human Subjects of the region Arnhem and Nijmegen approved the study. All participants gave their written informed consent.

### Study Design

During this cross-sectional study, participants underwent comprehensive vascular assessment including traditional measures (DC, cPWV, and IMT) and compound strain imaging.

## Vascular Measurements

All measurements were performed in supine position, in a temperature-controlled room, and following a ≥ 6 h fast, ≥18 h abstinence from caffeine, alcohol and vitamin supplements, and at least 24 h after strenuous physical activity by the participant. Measurements began after a resting period in the supine position for at least 15 min [20].

### Traditional vascular measurement: distensibility coefficient, central pulse wave velocity, and IMT

#### Distensibility coefficient

DC was assessed with a 9 MHz (L5–13) linear transducer connected to an Accuvix V10 ultrasound system (Samsung Medison, Seoul South Korea) in a transverse plane. Blood pressure was obtained in the right arm [21] and measured twice using a sphygmomanometer. Distensibility was measured twice in the left common carotid artery 1.5 and 2.0 cm upstream of the bulbous in the transverse plane.

#### Central pulse wave velocity

cPWV was measured using a three-lead electrocardiogram and an Echo-Doppler ultrasound machine (Waki Doppler, Atys Medical, Soucieu en Jarrest, France) at the left carotid artery and right common femoral artery [2]. The distances were measured between sternal notch and site of measurement for the carotid artery and common femoral artery via the umbilicus [2]. To estimate the travel distance between the carotid and femoral site, we subtracted the distance between the carotid location to the suprasternal notch from the distance between the suprasternal notch and the femoral site [22]. At least 10 cardiac cycles were recorded for the analysis.

#### Intima-media thickness

IMT of the left common carotid was recorded using a T3000 ultrasound system (Terason Teratech Corporation, Boston, United States) equipped with an 8.5 MHz 12 L5 linear transducer. To normalize vascular tonus the participants received 400 μg of sublingual glyceryl trinitrate (nitric oxide donor) before the IMT measurement [23]. Therefore, the IMT measurement took place after DC and compound strain imaging. Image sequences of ≥10 s were recorded 1.5 to 2.0 cm distally of the bifurcation of the common carotid, while having the vessel in a longitudinal imaging plane. Wall thickness were collected from two distinct angles and the mean value is presented.

### Compound strain imaging

Vascular radial strain was assessed with a 9 MHz (L5–13) linear transducer connected to an Accuvix V10 ultrasound system (Samsung Medison, Seoul South Korea) equipped with a dedicated multi-angle acquisition mode for compound strain imaging and with an interface providing ultrasound RF data. The subject was lying in supine position, with no pillow beneath the head. Blood pressure was manually measured using a sphygmomanometer in the right arm before each recording. Cumulative radial (RS) and circumferential (CS) strains from peak systole to end diastole were determined in the left common carotid. The end-diastolic phase was based on the carotid distention (pressure) waveform and defined as the time at which minimal lumen diameter was measured. The probe was placed at the location where the IMT was the thickest in the left common carotid artery. Whenever there was no location with a thicker IMT, the probe was placed between 1.5 and 2.0 cm upstream of the bulbous. Radiofrequency datasets of the carotid artery in a transverse plane were recorded twice for 3 seconds.

## Data Analysis

### Traditional vascular measurement: distensibility coefficient, cPWV, and IMT

DC was calculated from M-mode (Eq. 1) [2] and derived from a diameter curve that was obtained by manually indicating the lumen-intima interfaces for the near and far wall at peak systole, after which the software automatically traced the interfaces from systole to systole. DC values of measurement 1 and 2 were averaged per artery.

(1)distensibility $$ \frac{\mathrm{diameter}\kern0.2em {\mathrm{systole}}^2\kern0.2em {\textstyle \hbox{-}}\mathrm{diameter}\kern0.2em {\mathrm{diastole}}^2}{\mathrm{diameter}\kern0.2em {\mathrm{diastole}}^2\times PP} $$

(2) Pulse Pressure $$ P P=\mathrm{Systolic}\kern0.2em \mathrm{Blood}\kern0.2em \mathrm{Pressure}{\textstyle \hbox{-}}\mathrm{Diastolic}\kern0.2em \mathrm{Blood}\kern0.2em \mathrm{Pressure} $$

cPWV was calculated in Matlab (MATLAB and Statistics Toolbox Release R2014, The MathWorks, Inc., Natick, United States) and was based on the interval between the R-wave on the electrocardiogram and onset of the Doppler waveform. The onset of the Doppler waveform was semi-automatically detected by the software and only major deviations were operator corrected.

Intima-media thickness of the left common carotid artery was examined using custom-designed off-line edge-detection and wall-tracking software written in LabVIEW (LabVIEW 6.02, National Instruments, Austin, TX, USA). This DICOM-based software is largely independent of investigator bias and has been previously described in detail [24, 25]. Briefly, each recording was converted to a DICOM file at a frame rate of 30 Hz. Detection of the far wall media-adventitia interface was performed on every frame selected. The mean intima-media thickness was calculated via: (1/3 × systolic wall thickness) + (2/3 × diastolic wall thickness) [26]. All files were analyzed blinded by an independent researcher.

### Compound strain imaging

Strain parameters (RS and CS) were calculated from the radiofrequency data using custom made software written in Matlab R2014 (The MathWorks Inc., USA) [10, 19]. First ultrasonic frames were identified that corresponded to the systolic and diastolic phases (maximum and minimum lumen diameter, respectively) in an M-mode view of the image line that crossed the vessel lumen center. For each systolic frame a region-of-interest corresponding to the upper half segment of the vessel wall was manually selected for which strains were to be computed, tracked and accumulated from systole to diastole. Strain calculation was performed using a 2D cross-correlation based coarse-to-fine strain estimation strategy followed by displacement compounding, tracking, rotation and 2D least squares strain estimation, see Table 1 for detailed settings of the compound strain estimation [8, 10, 27, 28]. The algorithm provided strain values for every 62.5 μm (vertically) by 200 μm (horizontally) of tissue. Of all strain values in the region-of-interest, the 90th percentile of the maximal strain was defined as the final result of the strain analysis. Strain was normalized with respect to a reference pulse pressure of 40 mmHg. Strain values of measurement 1 and 2 were averaged.

**Table 1:** Settings of the strain estimation algorithm

	Iteration 1	Iteration 2	Iteration 3	Iteration 4^y^	Strain Estimation
Data input	Envelope	RF data	RF data	RF data	Radial displacements
Pre-kernel size (mm x mm)	1.3 × 0.6^a^	0.6 × 0.6^a^	0.3 × 0.6^a^	0.3 × 0.6^a^	-
Post-kernel size (mm x mm)	1.9 × 1.8^a^	0.9 × 1.8^a^	0.5 × 1.8^a^	0.5 × 1.8^a^	-
Axial kernel overlap (%)	80	80	80	80	-
Lateral kernel overlap (%)	67	67	67	67	-
Median filter size (mm x mm)	2.2 × 1.8^a^	1.1 × 1.8^a^	0.6 × 1.8^a^	0.9 × 0.6^az^ 0.6 × 0.6^az^	21° × 0.5 mm^x^
2D least squares strain estimator size (° x mm)	*-*	-	-	-	9° × 0.5 mm^x^

^a^Axial x lateral direction; y with sub-sample aligning (24); z-filter settings after compounding (15) for the cumulated horizontal and vertical displacements, respectively; x-filter sizes defined in circumferential x radial direction, because of conversion to polar grid with a spacing of 1° circumferentially and 100 μm radially (22)

## Statistical Analysis

This study was exploratory by design, since we had no information about RF-based strain in patients with cardiovascular diseases in non-stenotic arteries. Participants’ characteristics as well as vascular characteristics (DC, cPWV, IMT, RS, and CS) of the left common carotid of the participants were summarized with means and standard deviations or median and interquartile range (IQR). Parameters were checked for normality using a *Shapiro-Wilk* test and Q-Q plots. An independent Student’s *t* in case of normal distribution or *Mann-Whitney U* test for all other cases was used to analyse differences in participant characteristics and differences in compound strain imaging of non-stenotic carotid arteries between CVD and CON. To determine the coherence between traditional vascular measurements (DC, cPWV, IMT, RS, and CS) and compound strain imaging, correlation coefficients were calculated via *Spearman’s Rank* test for the total group and subgroups (CVD and CON). The Holm-Bonferoni method was used to correct for multiple pairwise testing [29]. Area under receiver operating characteristics (AROC)-curve was used to determine the discriminatory power of DC, cPWV, IMT, RS and CS. The different vascular measurements were combined to determine whether this increased the discriminatory power to detect CVD. All statistical analyses were performed using SPSS 22.0 software (IBM Corp. Released 2013. IBM SPSS Statistics for Windows, Version 22.0. Armonk, NY: IBM Corp.). Statistical significance was assumed at *p* < 0.05 (two-sided).

## Results

### Characteristics Participants

Age, weight, and body mass index did not significantly differ between CVD and CON (Table 2). CON were taller compared to CVD (CON: 180 ± 7 cm vs. CVD: 176 ± 6 cm; *P* = 0.011) and had a higher mean arterial pressure (CON: 100 mmHg [IQR: 93–106] vs. CVD: 94 mmHg [IQR: 90–100] mmHg; *P* = 0.035) (Table 2). Within the CVD group, anticoagulant (*n* = 35 [97%]) medications were used most frequently, followed by lipid lowering (*n* = 32 [89%]) and antihypertensive agents (*n* = 29 [81%]), whereas the control group did not use medications (Table 2).

**Table 2:** Participant characteristics of total and according to presence cardiovascular diseases

	Total group *n* = 72	Controls *n* = 36	CVD patients *n* = 36	*p* value
Characteristics				
Age (years)	60 ± 7	59 ± 7	61 ± 6	0.41
Body height (cm)	178 ± 7	180 ± 7	176 ± 6	0.011
Body mass (kg)	81 ± 11	81 ± 11	81 ± 12	0.98
Body Mass Index (kg/m^2^)^a^	25.3 [23.6 to 27.0]	24.9 [23.3 to 26.9]	25.6 [24.2 to 27.9]	0.20
Mean arterial pressure (mmHg)^a^	97 [91 to 105]	100 [93 to 106]	94 [90 to 100]	0.035
Diastolic blood pressure (mmHg)^a^	79 [75 to 88]	83 [77 to 91]	77 [73 to 82]	0.011
Systolic blood pressure (mmHg)	131 [123 to 142]	136 [124 to 142]	128 [120 to 139]	0.19
Cardiovascular medication use				
Anticoagulant (n)		-	35 (97%)	
Antihypertensive agents (n)		-	29 (81%)	
Lipid lowering agents (n)		-	32 (89%)	
Vascular measurements				
Radial strain (%)^a^	4.2 [3.2 to 6.5]	4.2 [3.2 to 6.4]	4.8 [3.6 to 6.5]	0.50
Circumferential strain (%)^a^	−10.0 [−13.6 to −6.5]	−10.2 [−14.9 to −7.0]	−8.8 [−13.2 to −6.2]	0.29
Distensibility^a^	0.27 [0.19 to 0.37]	0.27 [0.20 to 0.35]	0.29 [0.18 to 0.40]	0.77
Central pulse wave velocity^a^	8.0 [6.8 to 10.1]	7.8 [6.5 to 10.0]	8.0 [7.2 to 10.2]	0.22
Intima to media thickness (mm)^a^	0.72 [0.61 to 0.78]	0.68 [0.58 to 0.76]	0.76 [0.68 to 0.80]	0.023

*P*-value refers to an independent *Student’s t* or *Mann-Whitney U* test (two-sided) between controls and CVD patients. Data is presented as mean and standard deviation (normal distribution) or median and interquartile range (non-normal distribution)

^a^ *Mann-Whitney U* test

### Vascular Measures

The participants did not show visible atherosclerotic plaques during the vascular measurements. All compound strain measures took place approximately 1.5 to 2.0 cm below the bifurcation of the carotid artery. DC and cPWV did not significantly differ between CON and CVD (Table 2). CON demonstrated a smaller intima-media thickness of carotid artery compared to CVD (Table 2). RS and CS not significantly differ between CON and CVD, respectively (Table 2).

### Pairwise comparison: traditional measures vs. Compound strain imaging

Spearman rank analysis revealed that a higher CS or RS was associated with a higher DC and lower cPWV in the total group (Table 3). A higher DC was associated with a lower cPWV. IMT did not correlate with DC, cPWV, CS, and RS (Table 3).

**Table 3:** Spearman rank correlations between vascular measurements

	RS	CS	DC	cPWV	IMT	
RS		-0.72**	0.28	−0.34*	-0.05	CON
		-0.75**	0.23	−0.26	-0.15	CVD
CS	-0.73**		−0.34*	0.42*	0.07	CON
			-0.24	0.04	0.24	CVD
DC	0.27*	−0.32**		-0.26	0.14	CON
				-0.30	−0.17	CVD
cPWV	−0.25*	0.24*	−0.28*		0.28	CON
					-0.38*	CVD
IMT	-0.08	0.19	0.01	0.01		|

*RS* radial strain, *CS* circumferential strain, *DC* distensibility coefficient, *CON* controls, *CVD* patients with history of cardiovascular disease

*P < 0.05 **P < 0.01; numbers in bold remain statistical significant after correcting for multiple testing with the Holm-Bonferroni method [9]

### Area Under Receiver Operating Characteristic Curve

DC, cPWV, RS, and could not discriminate between CVD or CON (Table 4). The IMT was able to identify CVD with a best cut-off for the carotid IMT of 0.70 mm (sensitivity: 74%, specificity: 58%). Although combinations of IMT and RS, CS, DC, or cPWV increased the AUC, this was not statistically different from IMT only (Table 4).

**Table 4:** Area under the Receiver Operator Curve to determine the discriminatory power to detect patients with cardiovascular diseases for Radial Strain (RS), Circumferential Strain (CS), Distensibility Coefficient (DC), central Pulse Wave Velocity (cPWV), Intima-Media Thickness (IMT)

Parameter	AUC (95% CI)	P value
RS	0.57 (0.44 to 0.71)	0.28
CS	0.55 (0.41 to 0.68)	0.50
DC	0.48 (0.34 to 0.62)	0.76
cPWV	0.58 (0.45 to 0.72)	0.22
IMT	0.66 (0.53 to 0.79)	0.023
Combinations		
IMT + RS	0.64 (0.51 to 0.78)	0.04
IMT + CS	0.66 (0.53 to 0.79)	0.02
IMT + DC	0.65 (0.52 to 0.78)	0.03
IMT + cPWV	0.66 (0.53 to 0.79)	0.02
IMT + RS + CS	0.71 (0.59 to 0.84)	0.002
IMT + RS + CS + DC	0.71 (0.59 to 0.84)	0.002
IMT + RS + CS + DC + pPWV	0.71 (0.59 to 0.84)	0.003

*RS* radial strain, *CS* circumferential strain, *DC* distensibility coefficient, *IMT* intima-media thickness

## Discussion

The present study revealed that compound strain imaging is coherent to traditional measures of vascular stiffness (DC and cPWV). Compound strain imaging seems a feasible technique to measure carotid wall deformation, which are indirectly related to wall stiffness. However, compound strain imaging does not provide an incremental discriminative value to traditional measures of vascular health to discriminate between CVD and asymptomatic controls with non-stenotic arteries.

### Measuring Vascular Strain In Non-Stenotic Arteries

Strain estimation in the arterial wall is challenging because of the small structure of the arterial wall. Compound strain imaging was validated in severely stenotic arteries (>70% stenosis) [30] and demonstrated good correlation with local plaque composition. A larger area to calculate strain was present in these stenotic plaques than in the present study where none of the participants had severely stenotic arteries (IMT_total group_: 0.73 [0.61–0.78] mm), making a correct estimation of strain more challenging and probably more susceptible for errors. We can speculate that strain could provide additional information on changes of arterial elastic properties. The cPWV (carotid-fermoral) is considered the gold standard measurement to determine arterial stiffness [2]. Our results indicate that strain, DC, and cPWV are related measurements, since we observed a statistically significant correlation. Compound strain imaging seems technically feasible to measure in non-stenotic arteries. It is however questionable what additional information strain provides next to DC or cPWV when there is no or hardly any plaque present. Given the fact that compound strain imaging requires complex and intensive calculations, whereas DC and cPWV are relatively straightforward to determine and calculate, we would recommend using the DC or cPWV instead of strain to obtain an (indirect) measure for arterial stiffness in non-stenotic arteries.

### Vascular stiffness vs. Vascular structure

Elastic and structural properties of the arteries are influenced by different (lifestyle) factors [31–36]. In general, elasticity parameters seem to adjust in week-months by changing lifestyle (i.e.*,* physical activity) [35, 36] or medication usage [32]. Possibly, the reason why strain and distensibility could not discriminate between CVD or CON relates to the use of antihypertensive agents within the CVD group (Table 2). Antihypertensive agents are known to improve vascular compliance [31, 32], which may have reduced the arterial stiffness of the participants with CVD.

In the present study IMT was significantly larger among CVD patients compared to controls, despite that participants with CVD used (lipid lowering) medication. These results align with other studies, which demonstrated participants with prevalent CVD have an increased IMT compared to disease-free controls [37–40]. Lifestyle interventions (e.g.*,* diet, physical activity) and usage of lipid lowering agents following myocardial infarction reduces the progression of the IMT [33, 34]. IMT is therefore considered a more chronic stable index for the evaluation of vascular health. Care should be taken when evaluating vascular health in patients with CVD, since (lifestyle) interventions may reduce vascular stiffness, whereas structural measures may be better to evaluate long-term arterial properties and discriminate between chronically diseased and control participants.

### Limitations

A few methodological considerations should be taken into account to the present study. This study was cross-sectional by design and is subject to the inherent limitations of that approach. During this study, we could not evaluate the predictive value of compound strain imaging, since our CVD patient group already had the disease. Secondly, the offline analysis of the strain data is semi-automatic. The arterial wall was not always detected by the program and had to be retraced by the researcher. However, each measurement was evaluated by two researchers to determine whether the arterial wall was correctly traced. After consensus was met, the measurement was included in the statistical analysis. Third, DC was calculated using the pulse pressure of the right brachial artery instead of the local pulse pressure of the carotid artery. Due to pressure amplification [41] it is possible that we overestimated the pulse pressure in the carotid artery, since the amplitude of a pressure wave is higher in peripheral arteries than central arteries. However, with natural ageing the difference in arterial stiffness between central and peripheral arteries declines [41], which causes a fall in pressure amplification. Fourth, in the present study, we used a Doppler device to measure the cPWV. However, applanation tonometry is generally recognized as a more reliable technique to measure cPWV [42]. Fifth, in the past, we have gone through extensive efforts to improve the analysis [24, 25] and procedures to examine carotid IMT [23]. Nonetheless, we understand this approach may be slightly different compared to typically adopted protocols in the literature (including large cohort studies). We emphasize that, due to our protocol, generalizability against larger epidemiologic studies is inappropriate. Finally, all the participants of the study were men, which limits the generalizability of the present study.

## Conclusion

In non-stenotic arteries, compound strain imaging is a feasible technique to determine arterial wall deformation, but did not shown an incremental discriminative value next to DC, cPWV, or IMT in this study. This suggests that, in patients with non-stenotic arteries, compound strain imaging provides limited additional insight next to traditional measures of vascular health (DC, cPWV, or IMT) when differentiating between CVD patients and asymptomatic controls.

## Abbreviations

AROC: Area under Receiver operating characteristics
CON: Asymptomatic controls
CVD: Cardiovascular diseases
IMT: Intima-media thickness
IQR: Interquartile range
